# Supplementary figures and images for: Effects of quality-based procedure hospital funding reform in Ontario, Canada: An interrupted time series study
Source: PLoS One. 2020 Aug 19;15(8):e0236480. doi: 10.1371/journal.pone.0236480 (PMC7437861; doi:10.1371/journal.pone.0236480)

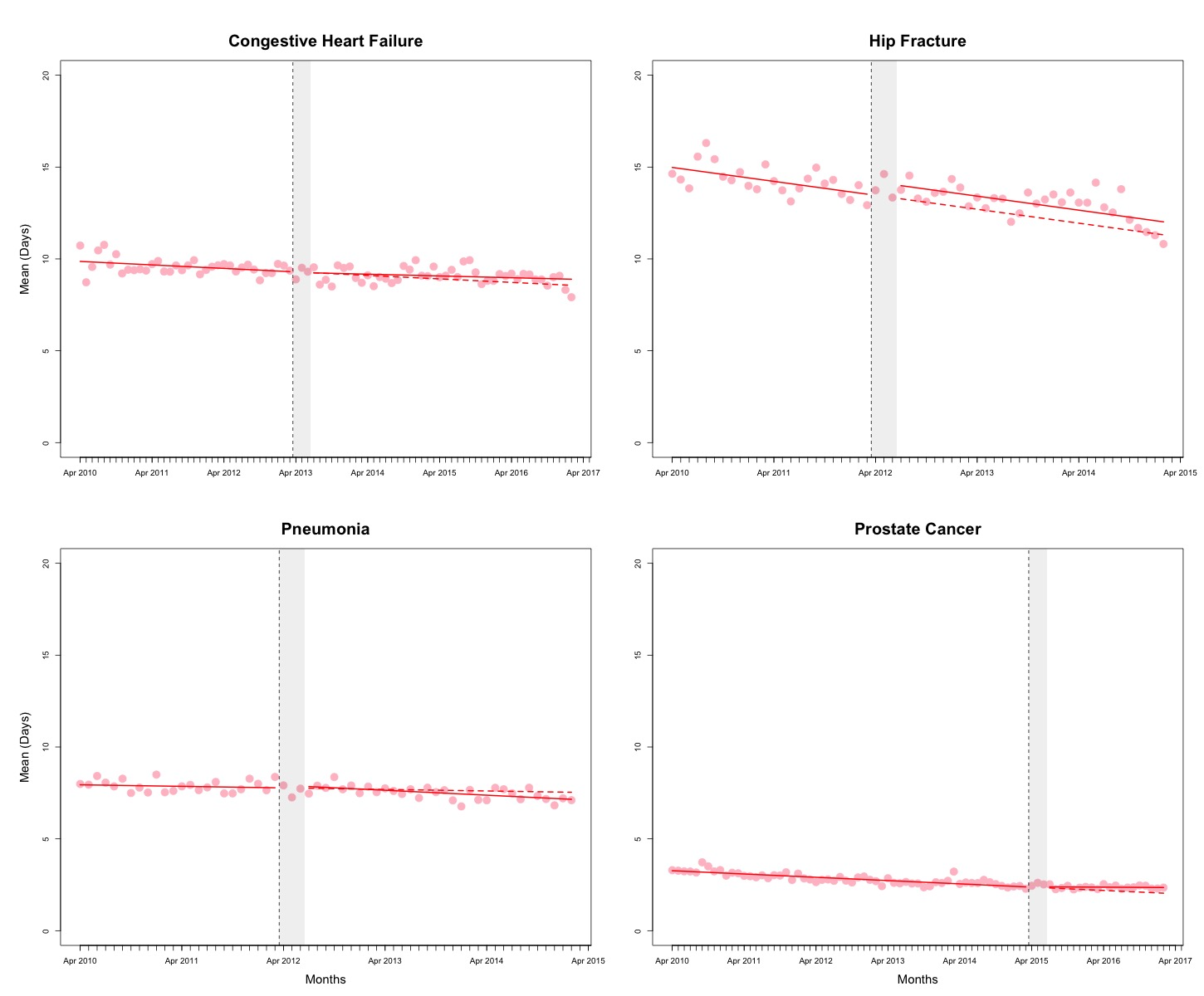

Supplement: S1 Fig — Red solid line represents the fitted model. The red dashed line represents the counterfactual (i.e. if no policy change occurred). The vertical dashed line represents the date of policy change. The grey shaded area represents the three months of “transition” period. Data are seasonally adjusted. (TIFF) [file pone.0236480.s001.tiff]

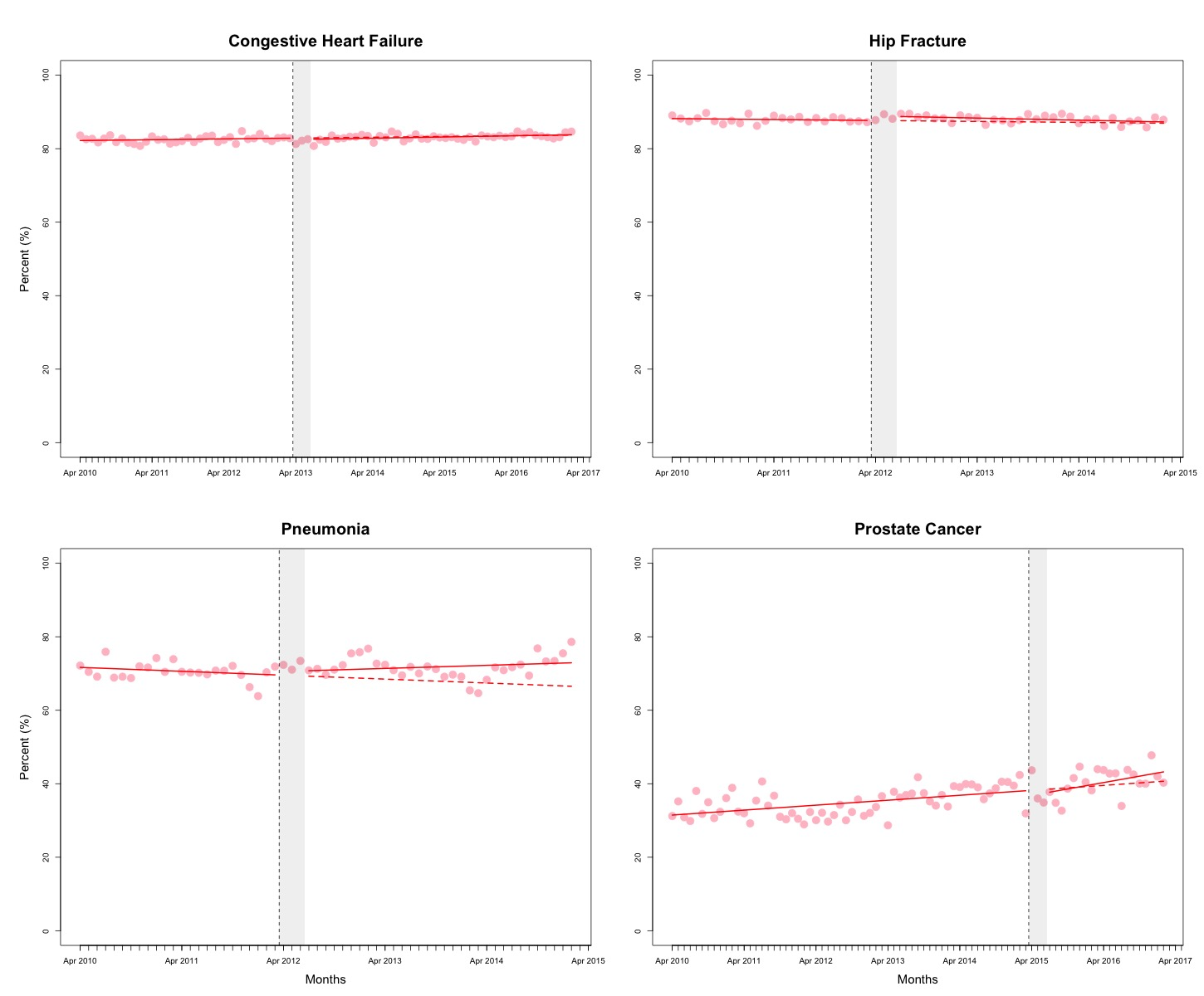

Supplement: S2 Fig — Red solid line represents the fitted model. The red dashed line represents the counterfactual (i.e. if no policy change occurred). The vertical dashed line represents the date of policy change. The grey shaded area represents the three months of “transition” period. Data are seasonally adjusted. (TIFF) [file pone.0236480.s002.tiff]

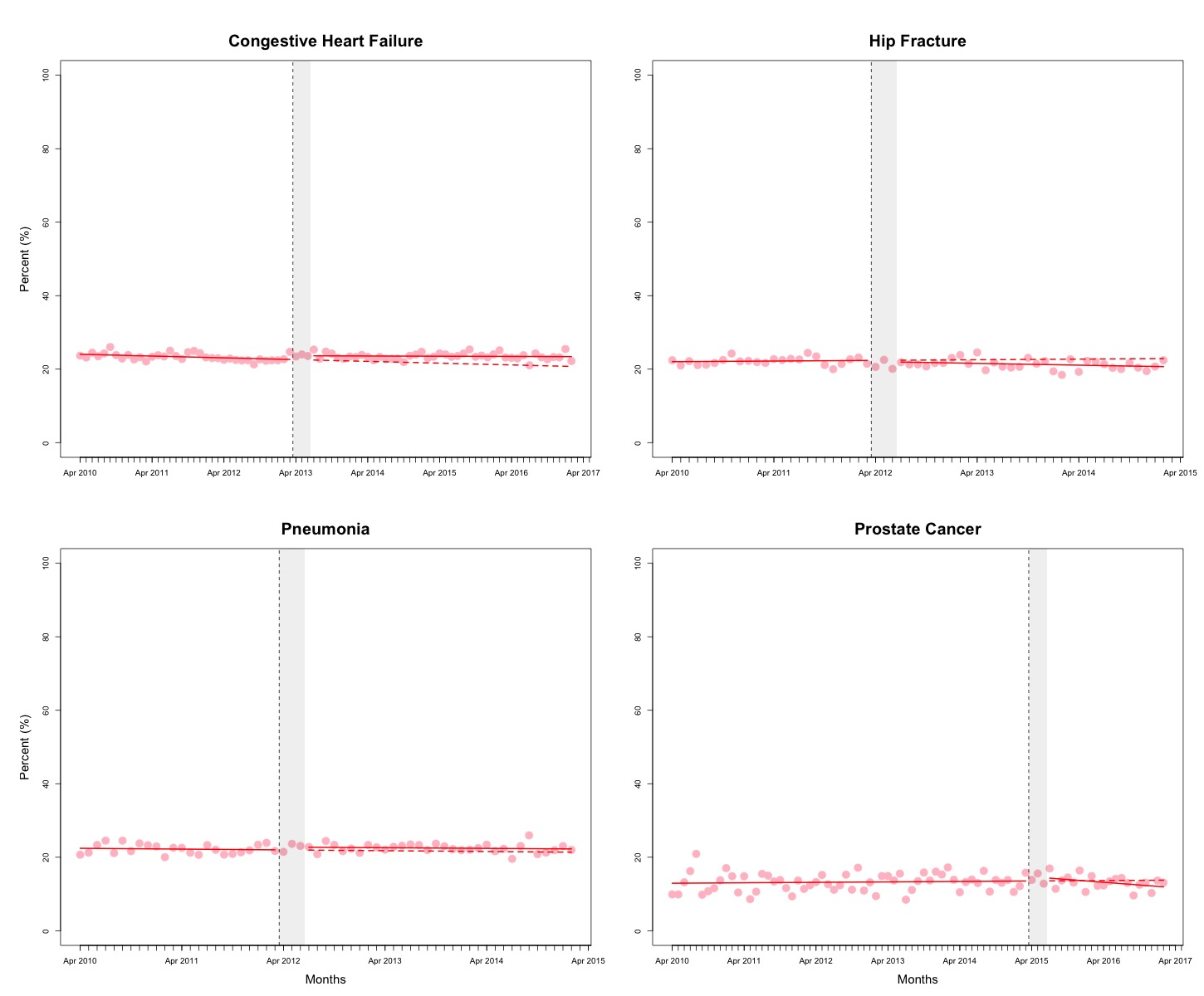

Supplement: S3 Fig — Red solid line represents the fitted model. The red dashed line represents the counterfactual (i.e. if no policy change occurred). The vertical dashed line represents the date of policy change. The grey shaded area represents the three months of “transition” period. Data are seasonally adjusted. (TIFF) [file pone.0236480.s003.tiff]

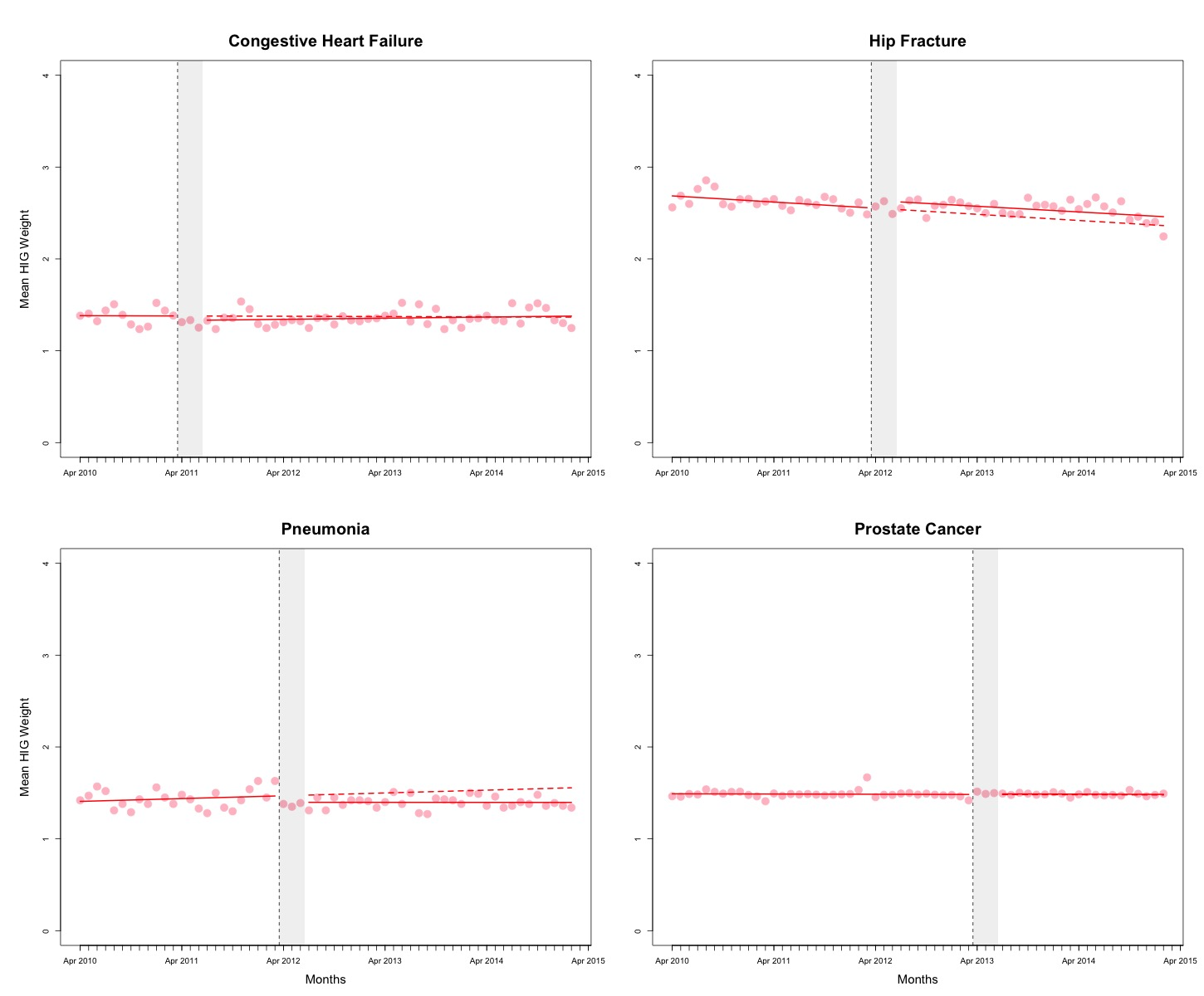

Supplement: S4 Fig — Red solid line represents the fitted model. The red dashed line represents the counterfactual (i.e. if no policy change occurred). The vertical dashed line represents the date of policy change. The grey shaded area represents the three months of “transition” period. Data are seasonally adjusted. (TIFF) [file pone.0236480.s004.tiff]

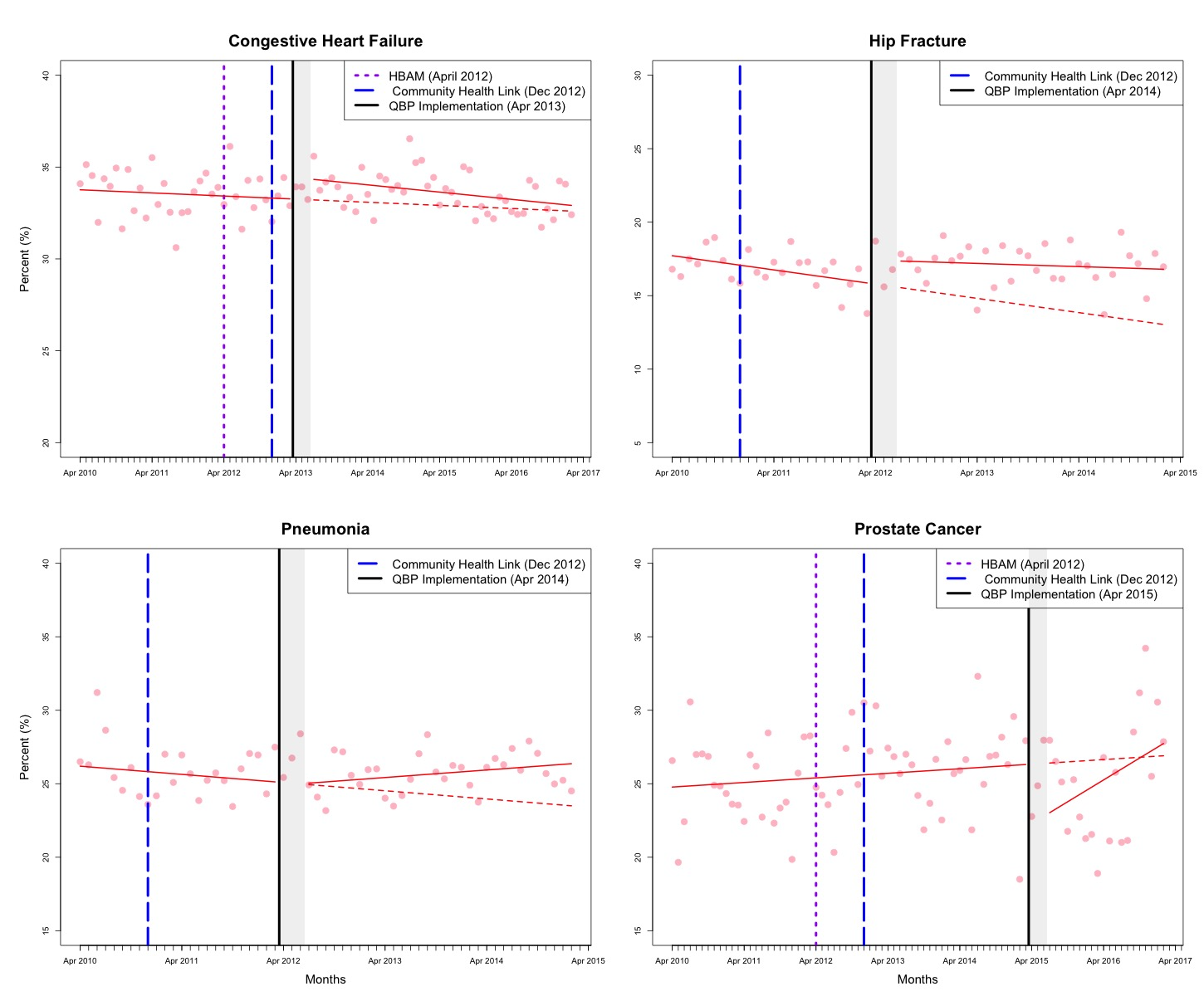

Supplement: S5 Fig — Red solid line represents the fitted model. The red dashed line represents the counterfactual (i.e. if no policy change occurred). The vertical dashed line represents the date of policy change. The grey shaded area represents the three months of “transition” period. Competing initiatives are outlined in the legend. Data are seasonally adjusted. (TIFF) [file pone.0236480.s005.tiff]

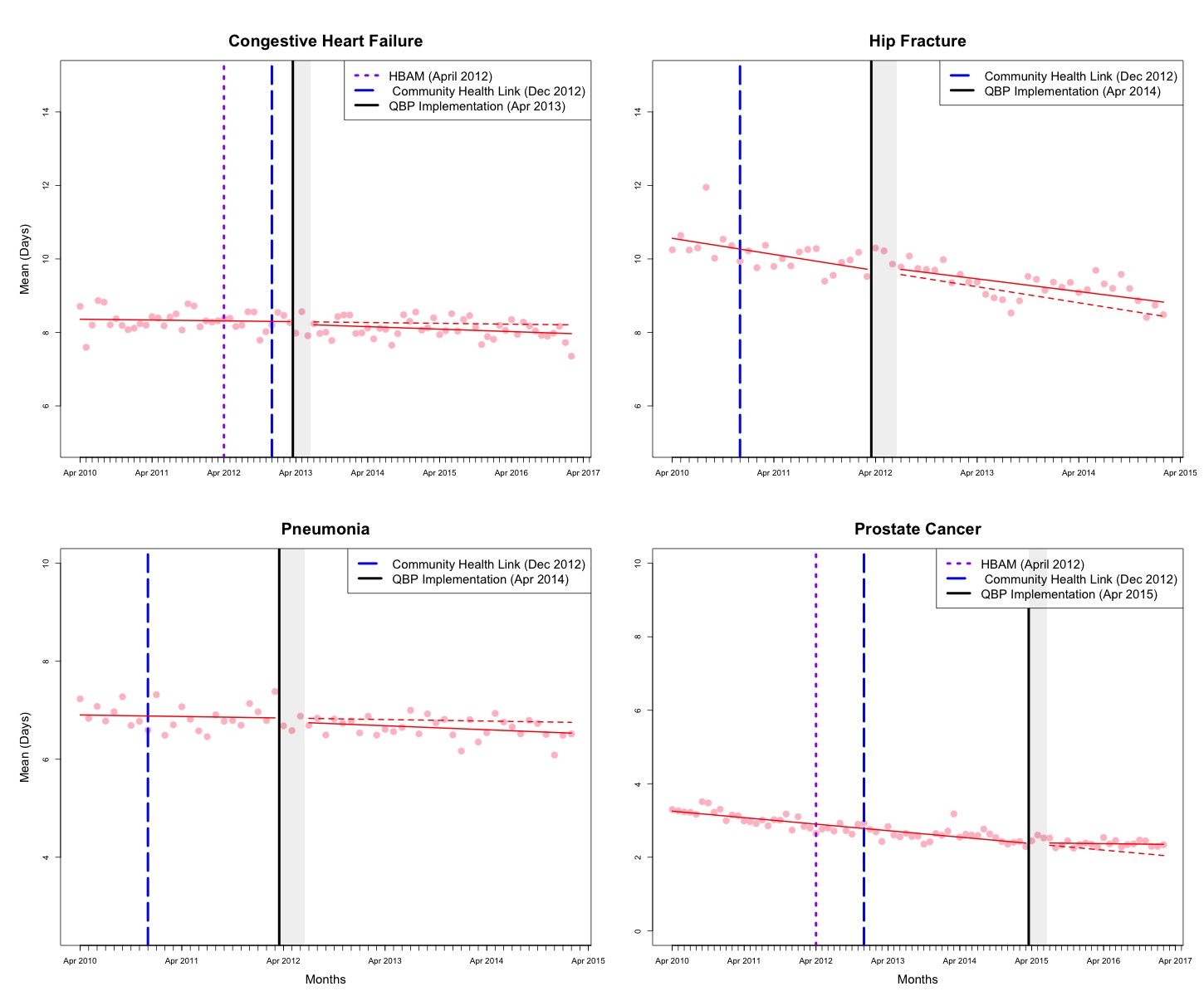

Supplement: S6 Fig — Red solid line represents the fitted model. The red dashed line represents the counterfactual (i.e. if no policy change occurred). The vertical dashed line represents the date of policy change. Competing Interventions are outlined in the legend. The grey shaded area represents the three months of “transition” period. Competing Initiatives are outlined in the legend. Data are seasonally adjusted. (TIFF) [file pone.0236480.s006.tiff]

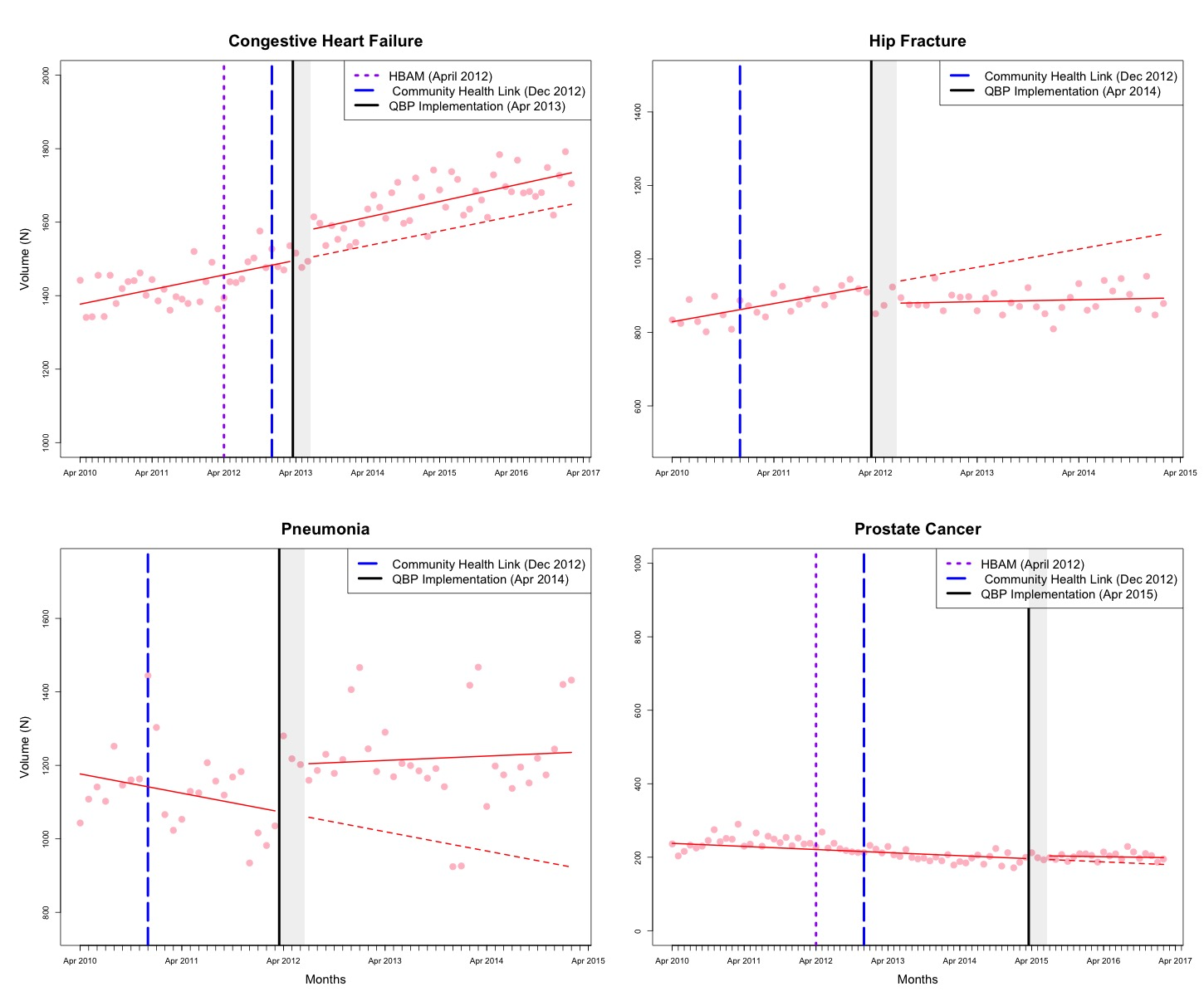

Supplement: S7 Fig — Red solid line represents the fitted model. The red dashed line represents the counterfactual (i.e. if no policy change occurred). The vertical dashed line represents the date of policy change. The grey shaded area represents the three months of “transition” period. Competing Initiatives are outlined in the legend. Data are seasonally adjusted. (TIFF) [file pone.0236480.s007.tiff]
