# Supplementary material for: Effects of quality-based procedure hospital funding reform in Ontario, Canada: An interrupted time series study
Source: PLoS One. 2020 Aug 19;15(8):e0236480. doi: 10.1371/journal.pone.0236480 (PMC7437861; doi:10.1371/journal.pone.0236480)
Supplement: S1 Table — (DOCX) [file pone.0236480.s008.docx]

**S1 Table: Cohort characteristics for Congestive Heart Failure patients included in the analysis**

|  | **2010** | **2011** | **2012** | **2013** | **2014** | **2015** | **2016** |
| --- | --- | --- | --- | --- | --- | --- | --- |
|  | N=17,833 | N=17,855 | N=18,709 | N=19,695 | N=20,933 | N=21,275 | N=19,623 |
| **Age (mean ± SD)** | 76.81 ± 12.56 | 76.88 ± 12.59 | 77.27 ± 12.62 | 77.38 ± 12.86 | 77.52 ± 12.89 | 77.54 ± 12.94 | 77.89 ± 12.79 |
| **Sex** |  |  |  |  |  |  |  |
| Male | 8,999 (50.5%) | 9,075 (50.8%) | 9,448 (50.5%) | 9,892 (50.2%) | 10,495 (50.1%) | 10,463 (49.2%) | 9,849 (50.2%) |
| **Neighbourhood income quintile*** |  |  |  |  |  |  |  |
| 1 (lowest) | 31,857 (23.4%) | 4,207 (23.6%) | 4,240 (23.7%) | 4,235 (22.6%) | 4,669 (23.7%) | 4,858 (23.2%) | 5,093 (23.9%) |
| 2 | 29,542 (21.7%) | 3,786 (21.2%) | 3,906 (21.9%) | 4,221 (22.6%) | 4,368 (22.2%) | 4,638 (22.2%) | 4,502 (21.2%) |
| 3 | 26,503 (19.5%) | 3,534 (19.8%) | 3,413 (19.1%) | 3,649 (19.5%) | 3,736 (19.0%) | 4,030 (19.3%) | 4,174 (19.6%) |
| 4 | 25,304 (18.6%) | 3,273 (18.4%) | 3,283 (18.4%) | 3,428 (18.3%) | 3,651 (18.5%) | 3,874 (18.5%) | 4,055 (19.1%) |
| 5 (highest) | 22,014 (16.2%) | 2,951 (16.5%) | 2,914 (16.3%) | 3,084 (16.5%) | 3,169 (16.1%) | 3,422 (16.3%) | 3,326 (15.6%) |
| **Living in a rural area*** | 12,399 (9.1%) | 1,664 (9.3%) | 1,567 (8.8%) | 1,700 (9.1%) | 1,821 (9.2%) | 1,981 (9.5%) | 1,938 (9.1%) |
| **Charlson Index (mean ± SD)** | 3.36 ± 1.95 | 3.42 ± 1.99 | 3.42 ± 1.98 | 3.41 ± 1.97 | 3.39 ± 1.97 | 3.34 ± 1.94 | 3.32 ± 1.93 |
| 0 | 672 (0.5%) | 94 (0.5%) | 86 (0.5%) | 80 (0.4%) | 110 (0.6%) | 102 (0.5%) | 109 (0.5%) |
| 1 | 27,844 (20.5%) | 3,495 (19.6%) | 3,546 (19.9%) | 3,718 (19.9%) | 3,948 (20.0%) | 4,341 (20.7%) | 4,475 (21.0%) |
| 2 | 18,412 (13.5%) | 2,555 (14.3%) | 2,427 (13.6%) | 2,567 (13.7%) | 2,678 (13.6%) | 2,845 (13.6%) | 2,791 (13.1%) |
| 3 | 32,087 (23.6%) | 4,076 (22.9%) | 4,095 (22.9%) | 4,257 (22.8%) | 4,634 (23.5%) | 4,995 (23.9%) | 5,168 (24.3%) |
| 4 | 24,256 (17.8%) | 3,118 (17.5%) | 3,151 (17.6%) | 3,428 (18.3%) | 3,501 (17.8%) | 3,712 (17.7%) | 3,810 (17.9%) |
| >=5 | 32,652 (24.0%) | 4,495 (25.2%) | 4,550 (25.5%) | 4,659 (24.9%) | 4,824 (24.5%) | 4,938 (23.6%) | 4,922 (23.1%) |
| **Number of emergency department visits in the past year (mean ± SD)** | 3.60 ± 3.53 | 3.53 ± 3.35 | 3.51 ± 3.17 | 3.59 ± 3.31 | 3.56 ± 3.24 | 3.64 ± 3.66 | 3.65 ± 4.09 |
| **Number of hospitalization days in the past year (mean ± SD)** | 13.13 ± 20.00 | 13.75 ± 21.41 | 13.61 ± 20.60 | 13.46 ± 20.53 | 13.20 ± 19.85 | 12.94 ± 19.56 | 12.66 ± 19.16 |
| **Facility type** |  |  |  |  |  |  |  |
| Community | 96,101 (70.7%) | 12,606 (70.7%) | 12,618 (70.7%) | 13,156 (70.3%) | 13,907 (70.6%) | 14,779 (70.6%) | 15,107 (71.0%) |
| Small | 373 (0.3%) | 48 (0.3%) | 50 (0.3%) | 55 (0.3%) | 60 (0.3%) | 58 (0.3%) | 52 (0.2%) |
| Teaching | 39,449 (29.0%) | 5,179 (29.0%) | 5,187 (29.1%) | 5,498 (29.4%) | 5,728 (29.1%) | 6,096 (29.1%) | 6,116 (28.7%) |
| Notes: * less than 1% missing data | | | | | | | |
