# Supplementary material for: Effects of quality-based procedure hospital funding reform in Ontario, Canada: An interrupted time series study
Source: PLoS One. 2020 Aug 19;15(8):e0236480. doi: 10.1371/journal.pone.0236480 (PMC7437861; doi:10.1371/journal.pone.0236480)
Supplement: S2 Table — (DOCX) [file pone.0236480.s009.docx]

**S2 Table: Cohort characteristics for hip fracture patients included in the analysis**

|  | **2012** | **2013** | **2014** | **2015** | **2016** |
| --- | --- | --- | --- | --- | --- |
|  | N=10,576 | N=11,289 | N=11,073 | N=10,876 | N=10,260 |
| **Age (mean ± SD)** | 80.24 ± 12.23 | 80.18 ± 12.17 | 80.53 ± 12.04 | 80.46 ± 12.17 | 80.16 ± 12.52 |
| **Sex** |  |  |  |  |  |
| Male | 3,212 (30.4%) | 3,478 (30.8%) | 3,446 (31.1%) | 3,363 (30.9%) | 3,205 (31.2%) |
| **Neighbourhood income quintile*** |  |  |  |  |  |
| 1 (lowest) | 2,330 (22.0%) | 2,525 (22.4%) | 2,402 (21.7%) | 2,312 (21.3%) | 2,142 (20.9%) |
| 2 | 2,136 (20.2%) | 2,208 (19.6%) | 2,180 (19.7%) | 2,202 (20.2%) | 2,118 (20.6%) |
| 3 | 2,045 (19.3%) | 2,259 (20.0%) | 2,188 (19.8%) | 2,095 (19.3%) | 2,045 (19.9%) |
| 4 | 2,029 (19.2%) | 2,107 (18.7%) | 2,236 (20.2%) | 2,146 (19.7%) | 2,034 (19.8%) |
| 5 (highest) | 1,988 (18.8%) | 2,121 (18.8%) | 2,010 (18.2%) | 2,052 (18.9%) | 1,859 (18.1%) |
| **Living in a rural area*** | 1,307 (12.4%) | 1,383 (12.3%) | 1,433 (12.9%) | 1,400 (12.9%) | 1,368 (13.3%) |
| **Charlson Index (mean ± SD)** | 1.36 ± 1.73 | 1.31 ± 1.70 | 1.36 ± 1.71 | 1.31 ± 1.66 | 1.26 ± 1.64 |
| 0 | 4,341 (41.0%) | 4,793 (42.5%) | 4,571 (41.3%) | 4,552 (41.9%) | 4,513 (44.0%) |
| 1 | 2,748 (26.0%) | 2,849 (25.2%) | 2,759 (24.9%) | 2,753 (25.3%) | 2,545 (24.8%) |
| 2 | 1,469 (13.9%) | 1,590 (14.1%) | 1,580 (14.3%) | 1,592 (14.6%) | 1,423 (13.9%) |
| 3 | 862 (8.2%) | 901 (8.0%) | 967 (8.7%) | 919 (8.4%) | 791 (7.7%) |
| 4 | 502 (4.7%) | 477 (4.2%) | 523 (4.7%) | 472 (4.3%) | 433 (4.2%) |
| >=5 | 654 (6.2%) | 679 (6.0%) | 673 (6.1%) | 588 (5.4%) | 555 (5.4%) |
| **Number of emergency department visits in the past year (mean ± SD)** | 2.29 ± 2.20 | 2.25 ± 2.18 | 2.36 ± 2.26 | 2.36 ± 2.60 | 2.33 ± 2.60 |
| **Number of hospitalization days in the past year (mean ± SD)** | 6.14 ± 14.69 | 5.72 ± 15.41 | 5.86 ± 13.72 | 5.75 ± 13.91 | 5.66 ± 14.27 |
| **Facility type** |  |  |  |  |  |
| Community | 7,756 (73.3%) | 8,283 (73.4%) | 8,096 (73.1%) | 8,057 (74.1%) | 7,670 (74.8%) |
| Small | 14 (0.1%) | 14 (0.1%) | 11 (0.1%) | 19 (0.2%) | 8 (0.1%) |
| Teaching | 2,806 (26.5%) | 2,992 (26.5%) | 2,966 (26.8%) | 2,800 (25.7%) | 2,582 (25.2%) |
| Notes: * less than 1% missing data, ^1^ | | | | | |
