# Supplementary material for: Effects of quality-based procedure hospital funding reform in Ontario, Canada: An interrupted time series study
Source: PLoS One. 2020 Aug 19;15(8):e0236480. doi: 10.1371/journal.pone.0236480 (PMC7437861; doi:10.1371/journal.pone.0236480)
Supplement: S3 Table — (DOCX) [file pone.0236480.s010.docx]

**S3 Table: Cohort characteristics for pneumonia patients included in the analysis**

|  | **2012** | **2013** | **2014** | **2015** | **2016** |
| --- | --- | --- | --- | --- | --- |
|  | N=14,375 | N=13,589 | N=15,431 | N=14,759 | N=13,587 |
| **Age (mean ± SD)** | 73.06 ± 17.30 | 73.16 ± 17.10 | 72.29 ± 17.49 | 73.70 ± 17.12 | 72.18 ± 17.71 |
| **Sex** |  |  |  |  |  |
| Male | 35,149 (49.0%) | 7,020 (48.8%) | 6,710 (49.4%) | 7,449 (48.3%) | 7,260 (49.2%) |
| **Neighbourhood income quintile*** |  |  |  |  |  |
| 1 (lowest) | 16,059 (22.4%) | 3,228 (22.5%) | 2,996 (22.0%) | 3,470 (22.5%) | 3,339 (22.6%) |
| 2 | 14,728 (20.5%) | 2,931 (20.4%) | 2,797 (20.6%) | 3,159 (20.5%) | 2,977 (20.2%) |
| 3 | 14,246 (19.9%) | 2,810 (19.5%) | 2,717 (20.0%) | 3,092 (20.0%) | 2,956 (20.0%) |
| 4 | 13,846 (19.3%) | 2,769 (19.3%) | 2,614 (19.2%) | 2,950 (19.1%) | 2,881 (19.5%) |
| 5 (highest) | 12,379 (17.3%) | 2,537 (17.6%) | 2,380 (17.5%) | 2,646 (17.1%) | 2,515 (17.0%) |
| **Living in a rural area*** | 6,800 (9.5%) | 1,445 (10.1%) | 1,270 (9.3%) | 1,440 (9.3%) | 1,414 (9.6%) |
| **Charlson Index (mean ± SD)** | 1.88 ± 2.00 | 1.88 ± 2.02 | 1.89 ± 2.01 | 1.87 ± 1.99 | 1.89 ± 2.01 |
| 0 | 22,568 (31.5%) | 4,518 (31.4%) | 4,240 (31.2%) | 4,806 (31.1%) | 4,701 (31.9%) |
| 1 | 15,394 (21.5%) | 3,201 (22.3%) | 2,950 (21.7%) | 3,349 (21.7%) | 3,046 (20.6%) |
| 2 | 12,294 (17.1%) | 2,362 (16.4%) | 2,274 (16.7%) | 2,694 (17.5%) | 2,561 (17.4%) |
| 3 | 8,497 (11.8%) | 1,678 (11.7%) | 1,613 (11.9%) | 1,828 (11.8%) | 1,750 (11.9%) |
| 4 | 5,033 (7.0%) | 989 (6.9%) | 952 (7.0%) | 1,114 (7.2%) | 1,054 (7.1%) |
| >=5 | 7,955 (11.1%) | 1,627 (11.3%) | 1,560 (11.5%) | 1,640 (10.6%) | 1,647 (11.2%) |
| **Number of emergency department visits in the past year (mean ± SD)** | 3.01 ± 3.96 | 2.92 ± 3.44 | 2.99 ± 3.45 | 3.00 ± 5.23 | 3.09 ± 3.91 |
| **Number of hospitalization days in the past year (mean ± SD)** | 8.59 ± 18.74 | 8.74 ± 18.80 | 8.71 ± 18.61 | 8.33 ± 18.18 | 8.52 ± 18.57 |
| **Facility type** |  |  |  |  |  |
| Community | 51,969 (72.4%) | 10,290 (71.6%) | 9,725 (71.6%) | 11,246 (72.9%) | 10,809 (73.2%) |
| Small | 283 (0.4%) | 73 (0.5%) | 61 (0.4%) | 59 (0.4%) | 50 (0.3%) |
| Teaching | 19,489 (27.2%) | 4,012 (27.9%) | 3,803 (28.0%) | 4,126 (26.7%) | 3,900 (26.4%) |
| Notes: * less than 1% missing data | | | | | |
