# Supplementary material for: Effects of quality-based procedure hospital funding reform in Ontario, Canada: An interrupted time series study
Source: PLoS One. 2020 Aug 19;15(8):e0236480. doi: 10.1371/journal.pone.0236480 (PMC7437861; doi:10.1371/journal.pone.0236480)
Supplement: S4 Table — (DOCX) [file pone.0236480.s011.docx]

**S4 Table: Cohort characteristics for prostate cancer patients included in the analysis**

|  | **2010** | **2011** | **2012** | **2013** | **2014** | **2015** | **2016** |
| --- | --- | --- | --- | --- | --- | --- | --- |
|  | N=2,897 | N=2,919 | N=2,707 | N=2,417 | N=2,329 | N=2,404 | N=2,252 |
| **Age (mean ± SD)** | 62.02 ±6.59 | 62.09 ± 6.46 | 61.93 ± 6.59 | 62.50 ± 6.70 | 62.98 ± 6.60 | 62.89 ± 6.75 | 63.37 ± 6.54 |
| **Sex** |  |  |  |  |  |  |  |
| Male | 2,897 (100.0%) | 2,919 (100.0%) | 2,707 (100.0%) | 2,417 (100.0%) | 2,329 (100.0%) | 2,404 (100.0%) | 2,252 (100.0%) |
| **Neighbourhood income quintile*** |  |  |  |  |  |  |  |
| 1 (lowest) | 376 (13.0%) | 364 (12.5%) | 357 (13.2%) | 342 (14.1%) | 303 (13.0%) | 331 (13.8%) | 284 (12.6%) |
| 2 | 557 (19.2%) | 485 (16.6%) | 484 (17.9%) | 423 (17.5%) | 450 (19.3%) | 394 (16.4%) | 402 (17.9%) |
| 3 | 605 (20.9%) | 580 (19.9%) | 523 (19.3%) | 480 (19.9%) | 442 (19.0%) | 491 (20.4%) | 434 (19.3%) |
| 4 | 612 (21.1%) | 706 (24.2%) | 626 (23.1%) | 540 (22.3%) | 491 (21.1%) | 565 (23.5%) | 555 (24.6%) |
| 5 (highest) | 743 (25.6%) | 774 (26.5%) | 711 (26.3%) | 629 (26.0%) | 636 (27.3%) | 619 (25.7%) | 576 (25.6%) |
| **Living in a rural area*** | 437 (15.1%) | 431 (14.8%) | 385 (14.2%) | 309 (12.8%) | 332 (14.3%) | 326 (13.6%) | 305 (13.5%) |
| **Charlson Index (mean ± SD)** | 2.31 ± 0.86 | 2.26 ± 0.79 | 2.28 ± 0.81 | 2.26 ± 0.75 | 2.30 ± 0.81 | 2.33 ± 0.91 | 2.38 ± 1.01 |
| 2 | 14,884 (83.0%) | 2,459 (84.9%) | 2,442 (83.7%) | 2,296 (84.8%) | 1,989 (82.3%) | 1,912 (82.1%) | 1,961 (81.6%) |
| 3 | 1,967 (11.0%) | 305 (10.5%) | 324 (11.1%) | 272 (10.0%) | 289 (12.0%) | 264 (11.3%) | 242 (10.1%) |
| 4 | 419 (2.3%) | 47 (1.6%) | 57 (2.0%) | 65 (2.4%) | 58 (2.4%) | 56 (2.4%) | 74 (3.1%) |
| >=5 | 655 (3.7%) | 86 (3.0%) | 96 (3.3%) | 74 (2.7%) | 81 (3.4%) | 97 (4.2%) | 127 (5.3%) |
| **Number of emergency department visits in the past year (mean ± SD)** | 0.40 ± 1.01 | 0.39 ± 0.91 | 0.43 ± 1.09 | 0.40 ± 0.92 | 0.40 ± 0.96 | 0.42 ± 1.08 | 0.40 ± 0.86 |
| **Number of hospitalization days in the past year (mean ± SD)** | 1.30 ± 1.77 | 1.29 ± 1.46 | 1.31 ± 1.64 | 1.33 ± 1.44 | 1.30 ± 1.31 | 1.35 ± 1.74 | 1.30 ± 1.78 |
| **Facility type** |  |  |  |  |  |  |  |
| Community | 1,698 (58.6%) | 1,829 (62.7%) | 1,517 (56.0%) | 1,306 (54.0%) | 1,282 (55.0%) | 1,317 (54.8%) | 1,233 (54.8%) |
| Teaching | 1,199 (41.4%) | 1,090 (37.3%) | 1,190 (44.0%) | 1,111 (46.0%) | 1,047 (45.0%) | 1,087 (45.2%) | 1,019 (45.2%) |
| Notes: * less than 1% missing data | | | | | | | |
