# Supplementary material for: Effects of quality-based procedure hospital funding reform in Ontario, Canada: An interrupted time series study
Source: PLoS One. 2020 Aug 19;15(8):e0236480. doi: 10.1371/journal.pone.0236480 (PMC7437861; doi:10.1371/journal.pone.0236480)
Supplement: S5 Table — (DOCX) [file pone.0236480.s012.docx]

**S5 Table : Results from segmented regression analysis of quality-based procedures on death or return to hospital, mean acute length of stay and volume**

|  | **Quality** | | **Access to Care** |
| --- | --- | --- | --- |
|  | Percent readmitted to hospital/ED or died  Estimate (95% CI) | Mean Acute Length of Stay  Estimate (95% CI) | Total volume  Estimate (95% CI) |
|  |  |  |  |
| **Congestive heart failure** |  |  |  |
| Intercept | 34.515 (33.781, 35.248) | 8.361 (8.146, 8.577) | 1374 (1336, 1411) |
| Pre-intervention slope^1^ | -0.014 (-0.049, 0.021) | -0.002 (-0.012, 0.008) | 3.314 (1.575, 5.053) |
| Level change^2^ | 1.135 (0.088, 2.181) | -0.076 (-0.380, 0.228) | 75.185 (22.947, 127.423) |
| Trend change^3^ | 0.01(-0.033, 0.053) | -0.004 (-0.016, 0.009) | 0.241 (-1.950, 2.433) |
| **Hip Fracture** |  |  |  |
| Intercept | 17.801 (16.756, 18.845) | 10.597 (10.218, 10.976) | 825 (809, 841) |
| Pre-intervention slope | -0.081 (-0.154, -0.008) | -0.037 (-0.063, -0.010) | 4.120 (2.909, 5.332) |
| Level change | 1.751 (0.273, 3.230) | 0.135 (-0.400, 0.670) | -56.826 (-80.259, -33.393) |
| Trend change | 0.063 (-0.025, 0.150) | 0.008 (-0.024, 0.039) | -3.689 (-5.005, -2.372) |
| **Pneumonia** |  |  |  |
| Intercept | 26.242 (25.375, 27.108) | 6.906 (6.744, 7.069) | 1181 (1049, 1314) |
| Pre-intervention slope | -0.046 (-0.108, 0.016) | -0.003 (-0.014, 0.009) | -4.37 (-13.574, 4.834) |
| Level change | 0.01 (-1.183, 1.204) | -0.084 (-0.313, 0.145) | 140.496 (-47.451, 328.442) |
| Trend change | 0.089 (0.017, 0.161) | -0.004 (-0.018, 0.009) | 5.36 (-5.650, 16.371) |
| **Prostate Cancer Surgery** |  |  |  |
| Intercept | 24.749 (22.748, 26.749) | 3.268 (3.159, 3.376) | 239 (205, 272) |
| Pre-intervention slope | 0.026 (-0.031, 0.083) | -0.015 (-0.018, -0.012) | -0.701 (-1.557, 0.155) |
| Level change | -3.602 (-7.628, 0.424) | 0.048 (-0.160, 0.255) | 9.418 (-21.529, 40.365) |
| Trend change | 0.222 (-0.068, 0.511) | 0.013 (-0.002, 0.028) | 0.467 (-2.279, 3.212) |
| ^1^ represents rate of change in outcome over time prior to QBP introduction  ^2^ represents an immediate increase or decrease following QBP introduction  ^3^ represents change in slope after QBP introduction relative to pre-intervention trend | | | |
