# Supplementary material for: Effects of quality-based procedure hospital funding reform in Ontario, Canada: An interrupted time series study
Source: PLoS One. 2020 Aug 19;15(8):e0236480. doi: 10.1371/journal.pone.0236480 (PMC7437861; doi:10.1371/journal.pone.0236480)
Supplement: S6 Table — (DOCX) [file pone.0236480.s013.docx]

**S6 Table. Results of segmented regression analysis of quality-based procedures on mean total length of stay, percent changes in patients over 65 and patients living in the lowest neighborhood income quintile and mean HIG weights**

|  | **Quality** | **Access to Care** | | **Coding behavior** |
| --- | --- | --- | --- | --- |
|  | Mean Total Length of Stay  Estimate (95% CI) | Percent of patients aged 65+  Estimate (95% CI) | Percent of Patients Living in the Lowest Neighborhood Income Quintile  Estimate (95% CI) | Mean HIG Weight  Estimate (95% CI) |
|  |  |  |  |  |
| **Congestive heart failure** |  |  |  |  |
| Intercept | 9.880 (9.586, 10.174) | 82.207 (81.645, 82.768) | 24.085 (23.429, 24.741) | 1.408 (1.312, 1.504) |
| Pre-intervention slope^1^ | -0.016 (-0.030, -0.002) | 0.024 (-0.003, 0.051) | -0.046 (-0.079, -0.013) | -0.005 (-0.018, 0.009) |
| Level change^2^ | -0.012(-0.426, 0.403) | -0.418(-1.228, 0.391) | 1.189 (0.205, 2.172) | -0.007 (-0.134, 0.121) |
| Trend change^3^ | 0.008(-0.009, 0.025) | 0.005(-0.028, 0.039) | 0.044 (0.003, 0.085) | 0.006 (-0.007, 0.020) |
| **Hip Fracture** |  |  |  |  |
| Intercept | 15.043 (14.272, 15.813) | 88.215 (87.450, 88.979) | 22.014 (21.009, 23.018) | 2.693 (2.606, 2.781) |
| Pre-intervention slope | -0.063 (-0.116, -0.010) | -0.021 (-0.075, 0.032) | 0.015 (-0.056, 0.085) | -0.006 (-0.012, 0.0004) |
| Level change | 0.718 (-0.365, 1.801) | 1.174 (0.093, 2.256) | -0.44 (-1.862, 0.982) | 0.083 (-0.041, 0.208) |
| Trend change | -0.0004 (-0.065, 0.064) | -0.026 (-0.090, 0.038) | -0.055 (-0.139, 0.029) | 0.001 (-0.007, 0.008) |
| **Pneumonia** |  |  |  |  |
| Intercept | 7.949 (7.809, 8.089) | 71.758 (68.118, 75.397) | 22.446 (21.410, 23.483) | 1.429 (1.364, 1.494) |
| Pre-intervention slope | -0.007 (-0.018, 0.004) | -0.089 (-0.339, 0.161) | -0.018 (-0.091, 0.054) | 0.001 (-0.004, 0.005) |
| Level change | 0.108 (-0.094, 0.311) | 1.325 (-3.807, 6.458) | 0.779 (-0.688, 2.245) | -0.036 (-0.128, 0.057) |
| Trend change | -0.015 (-0.027, -0.004) | 0.158 (-0.144, 0.461) | 0.003 (-0.083, 0.090) | -0.001 (-0.007, 0.004) |
| **Prostate Cancer Surgery** |  |  |  |  |
| Intercept | 3.281 (3.167, 3.395) | 31.363 (28.922, 33.805) | 12.910 (12.306, 13.514) | 238.503 (204.597, 272.409) |
| Pre-intervention slope | -0.015 (-0.018, -0.012) | 0.112 (0.044, 0.181) | 0.01 (-0.007, 0.028) | -0.701 (-1.557, 0.155) |
| Level change | 0.047 (-0.175, 0.269) | -1.016 (-5.446, 3.415) | 0.875 (-0.472, 2.222) | 9.418 (-21.529, 40.365) |
| Trend change | 0.013 (-0.003, 0.029) | 0.178 (-0.141, 0.497) | -0.134 (-0.240, -0.028) | 0.467 (-2.279, 3.212) |
| ^1^ represents rate of change in outcome over time prior to QBP introduction  ^2^ represents an immediate increase or decrease following QBP introduction  ^3^ represents change in slope after QBP introduction relative to pre-intervention trend | | | | |
