# Supplementary material for: Effects of quality-based procedure hospital funding reform in Ontario, Canada: An interrupted time series study
Source: PLoS One. 2020 Aug 19;15(8):e0236480. doi: 10.1371/journal.pone.0236480 (PMC7437861; doi:10.1371/journal.pone.0236480)
Supplement: S7 Table — (DOCX) [file pone.0236480.s014.docx]

**S7 Table: Overlapping Initiatives**

| Concurrent Initiative | Description |
| --- | --- |
| Excellent Care for All Act (2010) | This initiative was an important policy change, which was passed on 2010.  “The Act requires health care organizations, currently defined as hospitals, to: • Develop and post annual quality improvement plans. • Implement patient and employee satisfaction surveys and a patient relations process. • Link executive compensation to achievement of quality plan performance improvement targets. • Develop declarations of values after public consultation. • Create quality committees to report to each hospital board on quality related issues.”  Source: <https://www.ontariocanada.com/registry/view.do?postingId=4544&language=en> |
| Community Health Links, (2012) | This program provides individualized, coordinated, care plans for patients living with multiple chronic conditions and complex needs.  Source: <http://www.health.gov.on.ca/en/pro/programs/transformation/community.aspx> |
| Health Based Allocation Methods  (2012) | Health Based Allocation Methods (HBAM) was a new funding methodology to partially fund hospitals. HBAM uses expected weighted cases and expected unit cost.  Source: https://www.oha.com/Documents/HBAM-What%20You%20Need%20To%20Know.pdf |
| Austerity measures (since 2009) | “During a period of significant austerity beginning in 2009, Ontario’s hospitals contributed to getting the province back on track financially by accepting years of zero percent funding increases at a time when inflation, patient volumes, labour costs, energy, and regulatory requirements grew significantly”  More info: <https://www.oha.com/Bulletins/2558_OHA_A%20Sector%20on%20the%20Brink_rev.pdf> |
