# Supplementary material for: Effects of quality-based procedure hospital funding reform in Ontario, Canada: An interrupted time series study
Source: PLoS One. 2020 Aug 19;15(8):e0236480. doi: 10.1371/journal.pone.0236480 (PMC7437861; doi:10.1371/journal.pone.0236480)
Supplement: S1 File — (ZIP) [file pone.0236480.s015.zip › Li_DCP_QBP-ITS_2020Jun12_FINAL.pdf]

| Project Identification | |
|---|---|
| **ICES Project Title** | Interrupted time series analysis of the effects of hospital funding reforms in Ontario: a population-based retrospective cohort study |
| **ICES Project Number** | 2018 0990 037 000 |
| **Project Objectives** | *Insert Project Objectives as listed in the approved ICES Project PIA*<br>Evaluate the effects of QBPs on:<br>1) processes of care;<br>2) hospital admission and procedure volumes;<br>3) patient characteristics and outcomes; and<br>4) hospital discharge coding behaviour. |

| Cohort 1 of 4: Hip Fracture | |
|---|---|
| **Study Design** | ☒ Cohort study ☐ Matched cohort study ☐ Case-control study<br>☒ Cross-sectional study ☐ Other (specify): |
| **Index Event / Inclusion Criteria** | Index Event: Date of acute inpatient hospitalization for an incident hip fracture using CIHI-DAD as the data source.<br><br>Inclusion Criteria (adapted from Quality Indicators for Hip Fracture Quality-Based Procedures: Baseline Results. Ministry of Health and Long-Term Care, May 2015):<br>1. Ontario residents: Province_issuing_hcn= 'ON' or responsibility_for_payment='01'<br>2. Age greater than or equal to 18 y and < 105 y<br>3. Date of acute hospital admission 01/04/2012 to 28/02/2017<br>4. Most responsible diagnosis of hip fracture: Diag_code_01 = 'S720, 'S721', 'S722'<br>5. HBAM Inpatient Grouper (HIG): '726', '727', '766' (which were introduced April 2012)<br>6. First admission in episode is not QBP hospital<br>Exclude:<br>1. Most responsible diagnosis of Fracture of upper femoral epiphysis: Diag_code_01 = 'S7200'<br><br>If a patient was hospitalized for multiple hip fractures, include each hip fracture event except those with a previous hip fracture within 30 days. Please report the number of patients who had more than one hip fracture during the accrual period.<br><br>Include all eligible admissions/episodes [see outcome measures] regardless of facility and create a subset identified by the following QBP hospitals (NOTE: This corresponds with Facility from Instnum.xls):<br>882 699 800 664 763 888 704<br>650 596 826 804 931 916 940<br>928 726 793 640 619 907 814<br>967 968 890 707 965 852 661<br>974 745 995 966 905 753 665<br>970 813 858 718 898 701 674<br>842 957 777 935 959 606 955<br>947 693 950 953 736 954 632<br>930 952 941 960 771 933 942<br>975 951 936 962 958 976 927 906 |

| Cohort 1 of 4: Hip Fracture | | |
|---|---|---|
| **Estimated Size of Cohort (if known)** | MDRx of 11,556 in 2011/2012 | |
| **Exclusions (in order)** | *Step* | Description |
| | 1 | Invalid or missing IKN, date of birth, or sex |
| | 2 | Non-Ontario residents (use the “%getdemo” macro, the “prcddablk” variable, exclude recipients whose province code, pr, is not “35”) |
| | 3 | Age < 18 at index date in RPDB or age ≥105 years |
| | 4 | Exclude if “S72.00” |
| | 5 | Exclude if another index event occurred previously within 30 days of admdate (i.e. include only first event within 30 days period) |
| | 6 | First admission in episode of care was to a non-QBP hospital |

**Project Time Frame Definitions**

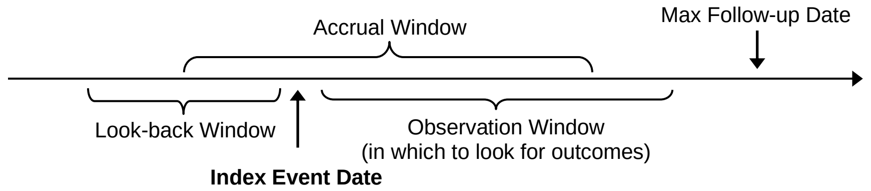


| | |
|---|---|
| **Accrual Start/End Dates** | *Accrual dates are based on the date of hip fracture hospitalization.*<br>Start: April 1, 2012 (when HIG were introduced)<br>End: February 28, 2017<br><br>Note: QBP implemented April 1, 2014 |
| **Max Follow-up Date** | March 31, 2017 |
| **When does observation window terminate?** | Follow subjects forward from the discharge date to the first of the following events:.<br>1. Unplanned admission to hospital/ED<br>2. 30 days since discharge date<br>3. Death |
| **Lookback Window(s)** | 3 years prior to admdate hip fracture hospitalization for co-morbid conditions (earliest potential date is April 1, 2007) *unless otherwise specified.* |
| | |

| Cohort 2 of 4: Pneumonia | |
|---|---|
| **Study Design** | ☒ Cohort study ☐ Matched cohort study ☐ Case-control study<br>☒ Cross-sectional study ☐ Other (specify): |
| **Index Event / Inclusion Criteria** | Index Event: Acute inpatient hospitalizations meeting the following entry criteria<br><br>Inclusion Criteria: Patients aged >=18 y assigned the following MRDx/DX10CODE1 in CIHI DAD from 01/04/2012 (when HIG were introduced) to 28/02/2017:<br>• J13 Pneumonia due to Streptococcus pneumonia<br>• J14 Pneumonia due to Haemophilus influenzae<br>• J15 Bacterial pneumonia, not elsewhere classified (NEC) |

| Cohort 2 of 4: Pneumonia | |
|---|---|
| | • J16 Pneumonia due to other infectious organisms, NEC<br>• J17.0 Pneumonia in bacterial diseases classified elsewhere<br>• J17.1 Pneumonia in viral diseases classified elsewhere<br>• J17.8 Pneumonia in other diseases classified elsewhere<br>• J18 Pneumonia, organism unspecified<br>• J10.0 Influenza with pneumonia, other influenza virus identified<br>• J11.0 Influenza with pneumonia, virus not identified<br>• J12 Viral pneumonia, NEC<br>(“J13”, “J14”, “J15”, “J16”, “J17.0”, “J17.1”, “J17.8”, “J18”, “J10.0”, “J11.0”, “J12”)<br><br>In addition, patients must fall within one of the following HIG (Health-Based Allocation Model Inpatient Groups):<br>138 Viral/unspecified pneumonia<br>136 Bacterial pneumonia<br><br>Include all eligible admissions regardless of facility and create a subset identified by the following QBP hospitals (NOTE: This corresponds with Facility from Instnum.xls):<br>882 699 800 664 763 888 704<br>650 596 826 804 931 916 940<br>928 726 793 640 619 907 814<br>967 968 890 707 965 852 661<br>974 745 995 966 905 753 665<br>970 813 858 718 898 701 674<br>842 957 777 935 959 606 955<br>947 693 950 953 736 954 632<br>930 952 941 960 771 933 942 961<br>975 951 936 962 958 976 927 906<br><br>NOTE: This cohort definition was taken from Quality-Based Procedures: Clinical Handbook for Community-Acquired Pneumonia, February 2014<br>http://www.health.gov.on.ca/en/pro/programs/ecfa/docs/qbp_pnemonia.pdf |
| **Estimated Size of Cohort (if known)** | ~58,000 |

| **Exclusions (in order)** | *Step* | Description |
|---|---|---|
| | 1 | Invalid or missing IKN, date of birth, or sex |
| | 2 | Non-Ontario residents (use the “%getdemo” macro, the “prcddablk” variable, exclude recipients whose province code, pr, is not “35”) |
| | 3 | Age < 18 at index date in RPDB or age ≥105 years |
| | Any of the following co-occurring diagnosis codes (types 1, 2, 3, W, X, or Y) | |
| | 4 | Pneumonia in Mycoses or Parasistic Diseases: “J17.2”, “J17.3” |
| | 5 | Aspiration Pneumonia: “J69.0”, “J69.1”, “J69.9” |
| | 6 | Neutropenia: “D70.0” |
| | 7 | HIV and AIDS: “B24”, “Z21” |
| | 8 | Bone Marrow Transplant “T86.000”, “T86.001”, “Z94.80”, “Z94.83” |

| Cohort 2 of 4: Pneumonia | | |
|---|---|---|
| | 9 | Systemic Chemotherapy: “Z51.1”, “Z54.2”, “Z92.6” |
| | 10 | Post-Transplant Immunosuppressive Therapy : “Z94.0”, “Z94.1”, “Z94.2”, “Z94.3”, “Z94.4”, “Z94.5”, “Z94.6”, “Z94.7”, “Z94.80”, “Z94.81”, “Z94.82”, “Z94.83”, “Z94.88”, “Z94.9”, |
| | 11 | Chronic Granulomatous Disease: “D71” |
| | 12 | Palliative Care: “Z51.5” |
| | Any of the following co-occurring CCI intervention codes | |
| | 13 | Bone marrow transplant: “1.WY.19.”, “1.LZ.19.HH-U7-A”, “1.LZ.19.HH-U7-J” |
| | 14 | Systemtic chemotherapy: “1ZZ.35.CA-M^”, “1.ZZ.35.HA-M^”, “1.ZZ.35.YA-M^” |
| | 15 | Exclude if another index event occurred previously within 30 days of admdate (i.e. include only first event within 30 days period) |

**Project Time Frame Definitions: Pneumonia**

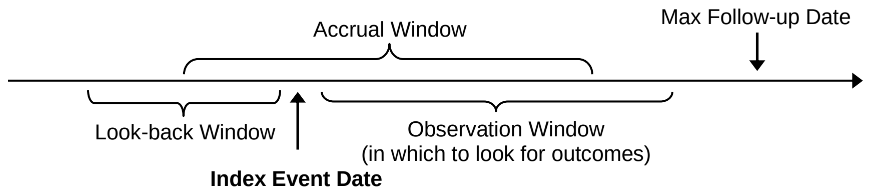


| | |
|---|---|
| **Accrual Start/End Dates** | *Accrual dates are based on the date of pneumonia hospitalization.*<br>Start: April 1, 2012<br>End: February 28, 2017<br><br>Note: QBP implemented April 1, 2014 |
| **Max Follow-up Date** | March 31, 2017 |
| **When does observation window terminate?** | Follow subjects forward from the discharge date to the first of the following events:.<br>1. Unplanned admission to hospital/ED<br>2. 30 days since discharge date<br>3. Death |
| **Lookback Window(s)** | 3 years prior to admdate for co-morbid conditions (earliest potential date is April 1, 2007) *unless otherwise specified.* |
| | |

| Cohort 3 of 4: Congestive Heart Failure | |
|---|---|
| **Study Design** | ☒ Cohort study ☐ Matched cohort study ☐ Case-control study<br>☒ Cross-sectional study ☐ Other (specify): |
| **Index Event / Inclusion Criteria** | Index Event: Acute inpatient hospitalizations meeting the following entry criteria<br><br>Inclusion Criteria: Patients aged >=20 y assigned the following MDRx/DX10CODE1 in CIHI DAD from 01/04/2010 to 28/02/2017. |

| Cohort 3 of 4: Congestive Heart Failure | |
|---|---|
| | • I50.x Heart failure, left ventricular dysfunction, etc.<br>• I40.x, I41.x Myocarditis<br>• I25.5 Ischemic cardiomyopathy<br>• I42.x, I43.x Cardiomyopathies<br>• I11.x plus I50.x Hypertensive heart disease plus heart failure, left ventricular dysfunction<br>• I13.x plus I50.x Hypertensive heart disease and renal disease plus heart failure, left ventricular dysfunction)<br><br>Reminder: Comorbidity diagnoses are those with diagnoses type "M" Most responsible, "1" pre-admission comorbidity, "2" post-admission comorbidity, or "W," "X," "Y" a service transfer diagnosis.<br><br>If a patient meets these criteria, include each event except those with a previous event within 30 days.<br><br>Include all eligible admissions regardless of facility and create a subset identified by the following QBP hospitals (NOTE: this corresponds with Facility from Instnum.xls):<br>882 699 800 664 763 888 704<br>650 596 826 804 931 916 940<br>928 726 793 640 619 907 814<br>967 968 890 707 965 852 661<br>974 745 995 966 905 753 665<br>970 813 858 718 898 701 674<br>842 957 777 935 959 606 955 949<br>947 693 950 953 736 954 632 731<br>930 952 941 960 771 933 942 961<br>975 951 936 962 958 976 927 906<br><br>NOTE: This cohort definition was taken from Quality-Based Procedures: Clinical Handbook for Heart Failure (Acute and Postacute), February 2015 http://www.health.gov.on.ca/en/pro/programs/ecfa/docs/qbp_heart.pdf |
| **Estimated Size of Cohort (if known)** | ~20,000 in 2011/2012 |

| **Exclusions (in order)** | *Step* | Description |
|---|---|---|
| | 1 | Invalid or missing IKN, date of birth, or sex |
| | 2 | Non-Ontario residents (use the "%getdemo" macro, the "prcddablk" variable, exclude recipients whose province code, pr, is not "35") |
| | 3 | Age < 20 at index date in RPDB or age ≥105 years |
| | 4 | MCC_PART = "I" |
| | 5 | Exclude if another index event occurred previously within 30 days of admdate (i.e. include only first event within 30 days period) |

| Project Time Frame Definitions: Congestive Heart Failure | |
|---|---|
| 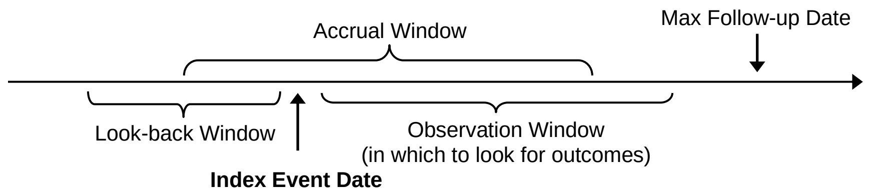 | |
| **Accrual Start/End Dates** | *Accrual dates are based on the date of CHF hospitalization.*<br>Start: April 1, 2010<br>End: February 28, 2017<br><br>Note: QBP implemented April 1, 2013 |
| **Max Follow-up Date** | March 31, 2017 |
| **When does observation window terminate?** | Follow subjects forward from the discharge date to the first of the following events:.<br>1. Unplanned admission to hospital/ED<br>2. 30 days since discharge date<br>3. Death |
| **Lookback Window(s)** | 3 years prior to admdate for co-morbid conditions (earliest potential date is April 1, 2007) *unless otherwise specified.* |

| Cohort 4 of 4: Prostate Cancer Surgery | |
|---|---|
| **Study Design** | ☒ Cohort study ☐ Matched cohort study ☐ Case-control study<br>☒ Cross-sectional study ☐ Other (specify): |
| **Index Event / Inclusion Criteria** | Index Event: will be defined as the date of acute inpatient hospitalization (MDRx)<br><br>Inclusion Criteria: All patients >=18 who were assigned as the MDRx in CIHI DAD from 01/04/2010 to 28/02/2017.<br><br>If a patient was hospitalized for multiple events, include each event except those with a previous event within 30 days. Please report the number of patients who had more than event during the accrual period.<br><br>We will include each patient with MRDx “C61” and a primary intervention [INCODE1] CCI code of “1QT91PB”, “1QT91PK”, “1QT91DA”; plus “IQT91DA” AND “7.SF.14.ZX”.<br><br>Include elective, urgent or emergent cases ADMCAT = “E”, “L”, “U”<br><br>Exclude:<br>- Interventions flagged as ‘Out of Hospital’: INOOH1=Y<br>- Interventions flagged as ‘Abandoned’: INATSTAT1=A (or GETDAD macro inatstatexcl=A)<br>- Interventions flagged as ‘Cancelled’: DX10CODE2-25=Z53<br><br>Include all eligible admissions from the following QBP hospitals (NOTE: This corresponds with Facility from Instnum.xls):<br>606 632 661 665 674 693 699 701 714 718<br>736 745 753 771 777 813 852 858 890 898<br>905 933 842 935 936 941 942 947 950 951 |

| Cohort 4 of 4: Prostate Cancer Surgery | |
|---|---|
| | 952 953 954 955 957 958 959 960 962 965<br>966 967 970 974 975 976 927 906 949 731<br><br>NOTE: See below for definition as appears in QBP manual |

| Factor | Included | Excluded |
|---|---|---|
| | A radical prostatectomy will be identified as a case that has:<br>- Main diagnosis code: C61 (malignant neoplasm of the prostate)<br>**AND**<br>Primary intervention field: one of the following CCI procedure codes<br>- 1QT91PB - Radical excision prostate (open perineal)<br>- 1QT91PK - Radical excision prostate (open retropubic)<br>- 1QT91DA – Radical excision prostate (laparoscopic)<br>Note: Robotic procedures are captured with the CCI procedure code 1QT91DA AND the subcode 7.SF.14.ZX Robotic assisted telemanipulation of tools, service, using system NEC, mandatory (robotic) | Records where main intervention is missing |
| Data Source | DAD | NACRS |
| Visit Type/ Activity | In-patient<br>• Elective cases<br>• Urgent cases<br>• Emergent cases | Day Surgery<br>• Interventions flagged as 'Out of Hospital'<br>• Interventions flagged as 'Abandoned'<br>• Interventions flagged as 'Cancelled |
| Additional Patient Factors | - Government insured patients only (i.e. OHIP)<br>- Patients 18 years of age and over | - Out-of-province records (i.e., Province not equal "ON")<br>- Records where responsibility for Payment is not |

| Cohort 4 of 4: Prostate Cancer Surgery | | | | |
|---|---|---|---|---|
| | | | | equal to '01'<br>- Records where calculated age is less than 18 years. Age is calculated as the difference between admit date and birth date |

| | | |
|---|---|---|
| **Estimated Size of Cohort (if known)** | ~23,000 | |
| **Exclusions (in order)** | *Step* | Description |
| | 1 | Invalid or missing IKN, date of birth, or sex |
| | 2 | Non-Ontario residents (use the "%getdemo" macro, the "prcddablk" variable, exclude recipients whose province code, pr, is not "35") |
| | 3 | Age < 18 at index date in RPDB or age ≥105 years |
| | 4 | Exclude if another index event occurred previously within 30 days of admdate (i.e. include only first event within 30 days period) |
| | 5 | Admissions to a non-QBP hospital |

**Project Time Frame Definitions: Prostate Cancer Surgery**

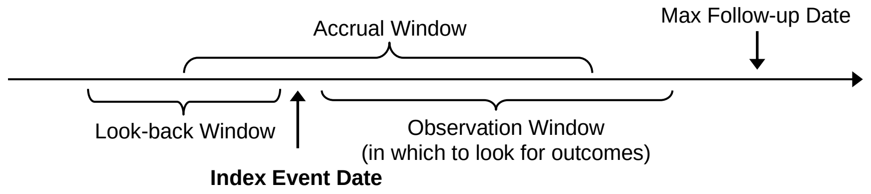


| | |
|---|---|
| **Accrual Start/End Dates** | *Accrual dates are based on the date of prostate cancer surgery*<br>Start: April 1, 2010<br>End: February 28, 2017<br><br>Note: QBP implemented April 1, 2015 |
| **Max Follow-up Date** | March 31, 2017 |
| **When does observation window terminate?** | Follow subjects forward from the discharge date to the first of the following events:.<br>1. Unplanned admission to hospital/ED<br>2. 30 days since discharge date<br>3. Death |
| **Lookback Window(s)** | 3 years prior to admdate for co-morbid conditions (earliest potential date is April 1, 2007) *unless otherwise specified.* |

**Variable Definitions (add additional rows as needed)**

| | |
|---|---|
| **Main Exposure or Risk Factor** | Month |

| Variable Definitions (add additional rows as needed) | |
|---|---|
| **Outcome Definition** | **<u>NOTE:</u>** These are monthly aggregated data<br><br>**<u>OBJECTIVE 1</u>**<br><br>**<u>Unplanned Return to ED/Hospital within 30 d (%)</u>**<br>**Include:**<br>➢ Unplanned admission to an acute care hospital within 30 days of discharge from index event<br>    o Unplanned admissions (ADMCAT ≠ L [elective])<br>➢ Unplanned admission to an ED within 30 days of discharge from index event<br>    o %GETNACRS inclscheduled = F, inclfrom_typee = T, inclto_typeip = T, inclnotseen = T<br>➢ Unplanned admission to either an acute care hospital or ED within 30 days of discharge from index event<br>**Exclude:**<br>➢ Transfers to another hospital; deaths in hospital and signouts: DISCHDISP =01, 03, 06, 07.<br><br>**<u>Mean and Median Acute Length of Stay for Hospital Episode or Admission</u>**<br>**Include:**<br>➢ ddate – admdate<br>**Exclude:**<br>➢ Transfers to another hospital; if DISCHDISP =01 *“ Transferred to another facility providing inpatient hospital care or acute care inpatient institution”*.<br><br>**<u>Mean and Median Total Length of Stay for Episode of Care</u>**<br>**Include visits with the same EPI:**<br>➢ Ddate(by EPI) – admdate<br><br>**<u>OBJECTIVE 2</u>**<br>**<u>Proportion over age 65</u>**<br>**Include:**<br>➢ Age in RPDB > 65<br><br>**<u>Proportion in lowest income quintile</u>**<br>**Include:**<br>➢ Incquint = “1” / Total number of patients; include missing values in incquint = “3”<br><br>**<u>OBJECTIVE 3</u>**<br>**<u>Volume</u>**<br>**Include:**<br>➢ # of admission episodes (monthly total)<br><br>**<u>OBJECTIVE 4</u>**<br>**<u>HIGWEIGHT</u>** |

| Variable Definitions (add additional rows as needed) | |
|---|---|
| | **Include:**<br>➢ 2017 HIG methodology regrouped HIGWEIGHT<br>o Monthly Mean, SD<br>o Monthly Median, IQR |
| **Baseline Characteristics** | **Observed at date of entry event unless otherwise indicated:**<br>- Age, y<br>- Sex<br>- Income quintile<br>- *if missing impute 3*<br>- Rural location<br>- *if missing impute urban*<br>- Deyo-Charlson Co-morbidity Index<br>- # of ER visits in the preceding year<br>- # of hospitalization days in the preceding year<br>- # (%) of patients admitted to QBP hospital<br>- QBP Hospital type (i.e. Academic, Community) |
